# Detecting Mitochondrial Oxygen Tension as a Measure of Local Tissue Oxygenation in Patients With Peripheral Arterial Disease Before and After Endovascular Therapy

**DOI:** 10.1177/15266028251344791

**Published:** 2025-06-17

**Authors:** Abdallah H. A. Zaid Al-Kaylani, Richte C. L. Schuurmann, Riemer H. J. A. Slart, Reinoud P. H. Bokkers, Jean-Paul P. M. de Vries

**Affiliations:** 1 Department of Radiology, Medical Imaging Center, University Medical Center Groningen, University of Groningen, Groningen, The Netherlands; 2 Division of Vascular Surgery, Department of Surgery, University Medical Center Groningen, University of Groningen, Groningen, The Netherlands; 3 Department of Nuclear Medicine and Molecular Imaging, Medical Imaging Center, University of Groningen, Groningen, The Netherlands

**Keywords:** peripheral arterial disease, endovascular therapy, perfusion, vasculature, mitochondrial oxygen tension

## Abstract

**Introduction:**

Assessment of mitochondrial oxygen tension (mitoPO_2_) is a novel technique for measuring skin perfusion. It is based on the oxygen-dependent quenching of delayed fluorescence of 5-aminolevulinic acid (5-ALA), known as the protoporphyrin IX-triple state lifetime technique. This study aimed to determine the tolerability and feasibility of measuring mitoPO_2_ in the lower limbs of patients with peripheral arterial disease (PAD) undergoing endovascular therapy. In addition, the study investigated the changes in mitoPO_2_ pre- and postoperatively.

**Materials and Methods:**

This prospective single-center study included patients with Rutherford stage 4 to 6 scheduled for endovascular therapy. Plasters containing 5-ALA were placed over the tibia and at the lower lateral leg 12 hours before the operation. 5-ALA tolerability was assessed by noting the occurrence of related side effects during application, measurements, and in the 48 hours after removal of the plaster. MitoPO_2_ was measured immediately before and after the operation over the tibia at the anterior tibialis muscle and the lateral side of the lower leg, and was followed by transcutaneous oxygen pressure and ankle-brachial index measurements.

**Results:**

Ten patients were included in this study. No side effects or adverse events related to 5-ALA were observed. One patient reported weak itching within 48 hours after removing the 5-ALA plaster. MitoPO_2_ measurements were feasible in all patients at the tibia and lower leg, but were not feasible on the dorsum of the foot. Postoperatively, a significant drop in mitoPO_2_ was detected at the tibia. No significant difference was found in mitoPO_2_ levels pre- and postoperative at the lower lateral leg. For transcutaneous oxygen pressure, no significant differences were detected postoperatively.

**Conclusions:**

5-ALA is tolerable and safe in patients with PAD. MitPO_2_ measurements at the tibia and lower lateral leg are feasible and capable of detecting changes in perfusion following endovascular therapy. Further research is needed with larger cohorts and longer follow-up to investigate the relationship between mitoPO_2_, oxygen supply, and tissue regeneration.

**Clinical Impact:**

This study demonstrated the feasibility and safety of mitochondrial oxygen tension (mitoPO_2_) measurement using 5-aminolevulinic acid (5-ALA) for assessing local skin perfusion in patients with peripheral arterial disease (PAD) undergoing endovascular therapy. Changes in mitoPO_2_ post-intervention suggest sensitivity to real-time microvascular and physiological alterations. This technique could potenitally improve overall patient outcomes and wound healing by enhancing patient stratification, treatment planning, perioperative monitoring, and postoperative follow-up.

## Introduction

Peripheral arterial disease (PAD) affects over 200 million people worldwide.^1,2^ Despite its widespread prevalence and serious consequences, PAD frequently goes unrecognized and inadequately treated.^3,4^ The main objective of PAD treatment is to improve blood flow with either surgical reconstruction or endovascular therapy (EVT).^5^ However, determining treatment endpoints remains challenging as evidenced by the high rates of re-obstructions and the need for reinterventions.^5–7^

The damage of PAD extends beyond the large blood vessels and also affects the microcirculation, which cannot be adequately assessed by common diagnostic tests like the ankle-brachial index (ABI).^8,9^ In addition, around 30% of patients have co-existing diabetes which exacerbates microvascular damage.^10,11^ Quantifying tissue perfusion and assessing the microvasculature could enhance PAD detection and evaluation, offer new endpoints for treatment guidance, and improve the prediction of treatment outcomes.^12,13^ The current gold standard for quantifying skin perfusion is transcutaneous oxygen pressure (TcPO_2_),^14^ which is a measure of microcirculatory levels of oxygen. However, it does not provide insight into intracellular metabolism and tissue utilization of oxygen.

The protoporphyrin IX (PpIX)-triplet state lifetime technique, developed by Mik et al, is a novel method for the noninvasive measurement of mitochondrial oxygen tension (mitoPO_2_) in human skin cells.^15–18^ To measure, 5-aminolevulinic acid (5-ALA) is topically applied to the area of measurement to enhance mitochondrial concentrations of PpIX. MitoPO_2_ is measured by exciting the PpIX and observing its delayed fluorescence. MitoPO_2_ represents the balance between cellular oxygen and demand,^19,20^ offering insights into local tissue perfusion and cellular oxygen metabolism. It remains unknown how PpIX is metabolized in the skin of PAD patients and whether they need a longer 5-ALA application time, or have a higher risk of developing adverse side effects as a result of their impaired skin perfusion.

This pilot study aimed to investigate the tolerability and safety of 5-ALA plasters and mitoPO_2_ measurements in patients with PAD undergoing EVT. Additionally, the feasibility and reliability of obtaining mitoPO_2_ measurements pre- and post-EVT were assessed. Secondary objectives included determining the optimal anatomical site for mitoPO_2_ measurement by comparing signal quality across different locations, evaluating the impact of different plaster application times (12 hours vs 20 hours) on signal reliability, and comparing mitoPO_2_ measurements with conventional perfusion markers, such as TcPO_2_ and ABI. Furthermore, we explored whether mitoPO_2_ measurements were sensitive to detect acute perfusion changes following EVT and investigated the possibility of reusing 5-ALA plasters without compromising data quality.

## Materials and Methods

This study was a single-center prospective cohort study, in which 10 patients with PAD and scheduled for EVT at the University Medical Centre Groningen (UMCG) were enrolled from April 2022 to October 2023. The study protocol was approved by the Institutional Review Board (IRB) of the UMCG (IRB number 2021/539) and was registered under the European Union Clinical Trials Register (EudraCT No. 2021-004870-78). Written informed consent was obtained from all patients.

The study was carried out according to the Medical Research Involving Human Subjects Act and the Declaration of Helsinki. The STROBE (Strengthening the Reporting of Observational Studies in Epidemiology) guidelines^21^ were used when reporting the methodology and results of this study.

### Study Population

Patients diagnosed with PAD and a scheduled EVT were included in this study. The Rutherford classification and treatment plan were determined by a multidisciplinary team consisting of interventional radiologists and vascular surgeons based on clinical evaluation and imaging. Inclusion criteria were age >50 years, Rutherford classes 4 to 6, and without ulcers or tissue damage in the areas of perfusion measurements with the COMET and TcPO_2_ devices. Exclusion criteria included recent lower leg fractures, (partial) leg amputations, tattoos in the location of the plasters, known allergy to the excipients of the 5-ALA plasters, known diagnosis of porphyria or photodermatoses, previous photodynamic therapy or recent use of tanning bed, use of medicinal products with known phototoxic or photoallergic potential which may enhance the phototoxic reaction, and use of other topical medicinal products at the same time of 5-ALA application and a language barrier. Baseline patient characteristics, Rutherford classification, and procedural details were collected from medical records.

### Mitochondrial Oxygen Tension, Consumption, and Delivery

**Figure 1.**
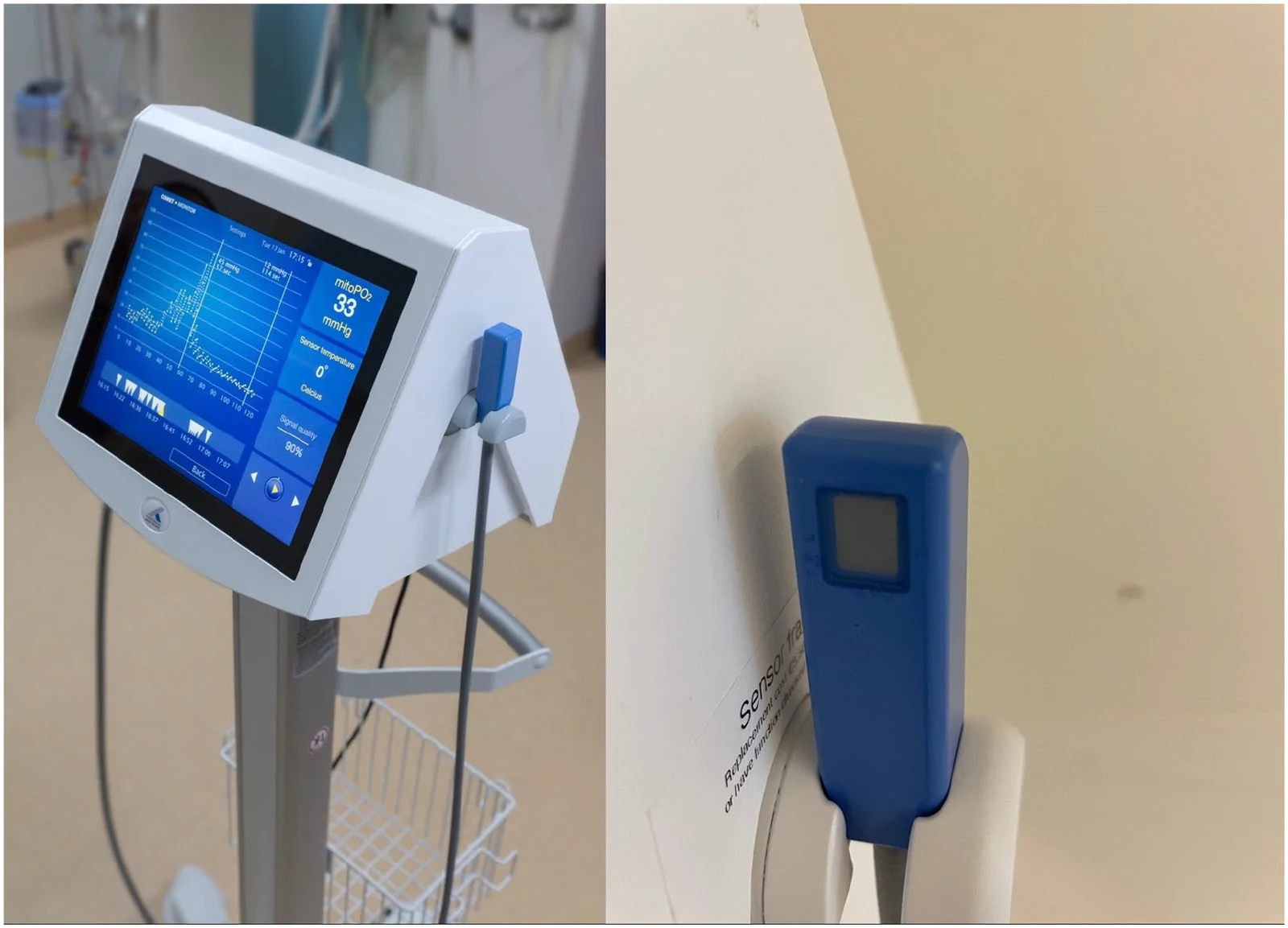
COMET measurement system (left) and COMET skin sensor (right).

MitoPO_2_ can be measured using the COMET system (Photonics Healthcare B.V., Utrecht, The Netherlands) which consists of 2 main components: the COMET measurement device and a skin sensor (Figure 1). To measure, the skin is first primed with 5-ALA using transdermal plasters of Alacare (Photonamic GmbH & Co. KG, Pinneberg, Germany). This is done to enhance intracellular PpIX concentrations^22^ and ensure that the signal originates from mitochondria.^15,23,24^ The COMET employs a pulsed laser to generate short (<100 nseconds) pulses of light (515 nm) that induce photo-excitation in the accumulated PpIX, leading to delayed fluorescence, which is measured with the sensor. The relationship between the lifetime of this fluorescence is inversely related to the amount of mitoPO_2_,^15^ and can be expressed using the Stern–Volmer equation:

$${\mathrm{mitoPO}}_{2}=\left(\frac{1}{t}-\frac{1}{t_{0}}\right)/k_{q}$$

in which t is the lifetime of the delayed fluorescence, $k_{q}$ represents the quenching constant, and $t_{0}$ is the lifetime of the delayed fluorescence in the absence of oxygen.^17^ The COMET system measures in a roughly circular area of about 5 mm^2^ in the epidermis with a thickness of 0.1 mm,^25^ resulting in a measurement volume of around 0.5 mm^3^.^26^

In addition to mitoPO_2_, it is possible to measure mitochondrial oxygen consumption (mitoVO_2_)^17^ and delivery (mitoDO_2_).^27^ MitoVO_2_ is measured by determining the oxygen disappearance rate (ODR).^28^ To determine the ODR, local occlusion of the circulation is induced through the application of manual pressure on the COMET skin sensor. This allows the cessation of microvascular oxygen supply without disrupting cellular consumption.^28^ MitoVO_2_ is then calculated from the descending slope. After the release of the manual pressure, mitoDO_2_ can be calculated from the ascending slope. To facilitate this, dynamic measurements were utilized in which 120 measurements of mitoPO_2_ were acquired at a frequency of 1 measurement/second.

### Measurement Protocol

**Figure 2.**
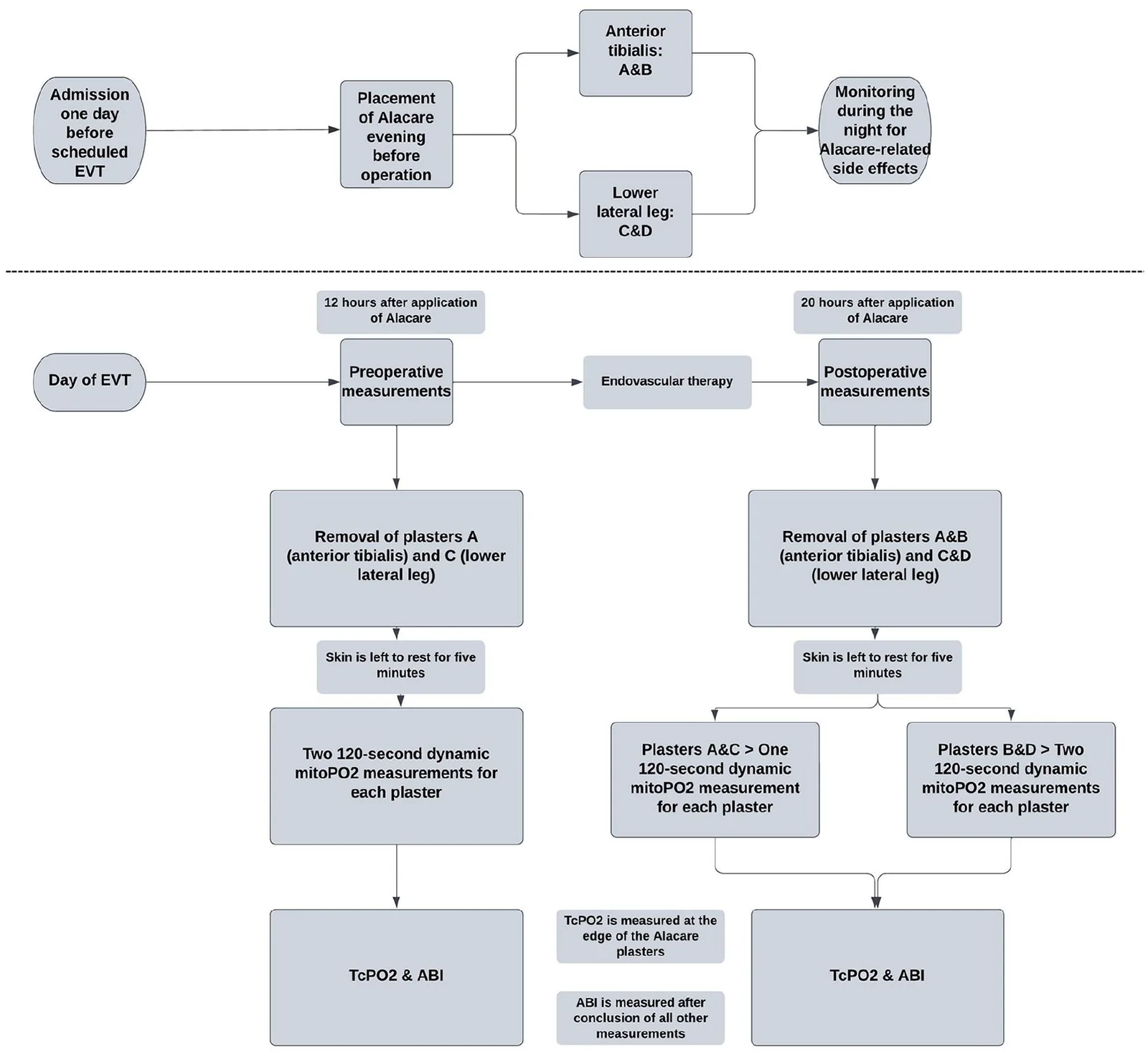
Flowchart of the measurement protocol.

MitoPO_2_ measurements were performed using a standardized measurement protocol (Figure 2). All measurements took place in the surgical ward. Patients were admitted 1 day before their scheduled EVT.

**Figure 3.**
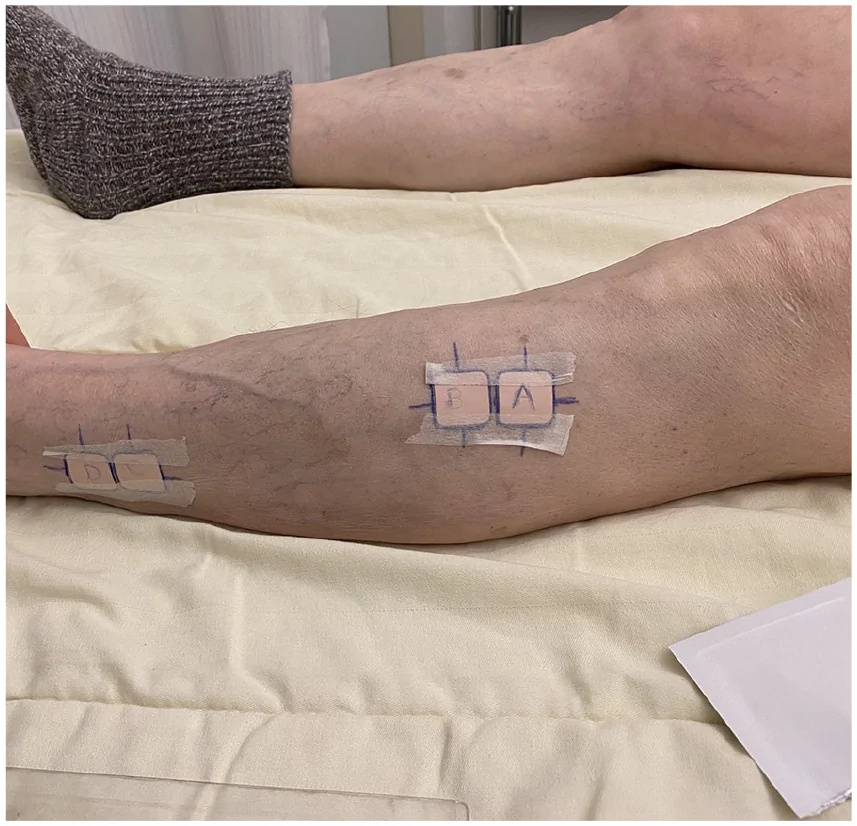
Alacare plasters at the measurement locations. Plasters A and B were placed at the anterior tibialis muscle, whereas plasters C and D were placed at the lower lateral leg. Plasters A and C were used in the pre- and postoperative measurement sessions, while B and D were only used for the postoperative session to test whether plaster reuse affected mitoPO_2_ values in the postoperative measurements.

To ensure sufficient signal quality, the 5-ALA plasters were applied in the evening prior to the EVT. On the day of the EVT, participants underwent 2 measurement sessions; 1 before the EVT and 1 after. The timing of the measurements was standardized for all patients, respectively, 12 and 20 hours after 5-ALA application for the pre- and post-EVT measurements. Two plasters (A and B) were placed at the middle of the tibia over the anterior tibialis muscle, and 2 more (C and D) were positioned on the lateral side of the leg, 10 cm above the lateral malleolus (Figure 3). In the pre-EVT session, plasters A and C were used as measurement locations. In the post-EVT session, all plasters (A, B, C, and D) were used to investigate the effect of plaster reuse. Initially, the second location was intended to be the dorsum of the feet. However, these measurements proved to be not feasible due to difficulties in placing the COMET skin sensor. Consequently, after the first 4 patients, the location was changed to the lateral lower leg.

All patients rested in the supine position for 15 minutes before the beginning of the measurement sessions. Pre- and post-EVT measurements included mitoPO_2_, TcPO_2_, and ABI measurements. First, the 5-ALA plaster was first removed, and the area was covered and left to rest for 5 minutes, during which the sensor was warmed to skin temperature. After 5 minutes, the sensor was placed on the measurement area, followed by 2 dynamic measurements of 120 seconds each. The first measurement determined the average mitoPO_2_ and signal quality, while the second involved inducing local pressure for the calculation of mitoVO_2_ and mitoDO_2_. This process was repeated for all measurement locations. Following the preoperative mitoPO_2_ measurements, the 5-ALA plasters were reapplied. TcPO_2_ measurements were then conducted medial to and at the edge of the 5-ALA plasters for comparability. ABI was measured after completing all other measurements. At the end of both measurement sessions, all 5-ALA plasters were removed, and the skin was covered with an elastic wound patch to protect against ambient light. Patients were instructed to keep the patch on for 48 hours.

### Treatment and Clinical Outcomes

Patients were treated in accordance with the European guidelines for clinical care.^29^ All lesions were treated with percutaneous transluminal angioplasty (PTA) or PTA with additional stent placement in case of >30% residual stenosis measured on angiography, flow-limiting dissections, or acute recoil.^29^ The technical success of the treatment was determined by the operating interventionist, defined as having less than 30% residual stenosis on the completion angiogram.^30^ The Global Limb Anatomic Staging System (GLASS) classification was used by the interventionist to score the treated lesions.^5^

Clinical outcomes were assessed during the first follow-up visit post-EVT in the outpatient clinic by a vascular surgeon. Clinical improvement was characterized by a reduction of at least one Rutherford stage. Moreover, for patients with Rutherford stages 5 to 6, an assessment of wound surface area was conducted by a wound care nurse and a vascular specialist, comparing it to the pre-EVT measurements. Improvement was defined as a decrease in wound surface area or complete healing.

### Tolerability of 5-ALA

The tolerability of the 5-ALA was assessed by noting the occurrence and severity of itching, pain, burning sensation, irritation, and local redness at the location of the plasters. The verbal rating scale^31^ was used to assess itching, while the other side effects were assessed visually and via standardized questionnaires. Tolerability was assessed in 3 instances, namely during the 5-ALA application, during mitoPO_2_ measurements, and in the 48 hours after the removal of the plasters.

### Feasibility of mitoPO_2_ Measurements

The feasibility of mitoPO_2_ measurements was assessed by the display of the measurements on the COMET monitor with sufficient signal quality. As described by Ubbink et al,^26^ the quality of the signal is determined based on the signal-to-noise ratio (SNR) value, with each unit increase in SNR correlating to an approximate 1% improvement in signal quality up to an SNR level of 50. Measurements with an SNR above 20 are considered reliable.^26^ Alacare plasters have an estimated minimum application time of 4 hours,^32^ but studies have obtained reliable measurements with application times ranging between 5 and 17 hours.^33^ Due to the lower perfusion in patients with PAD, the process might be slower and therefore, the signal quality might need more time to reach the required level for accurate measurement of mitochondrial oxygenation. Therefore, to ensure the reliability of the measurements, the plasters in this study were applied for approximately 12 and 20 hours for the pre- and post-EVT measurements, respectively. Additionally, plaster reusability was investigated by comparing signal quality and mitoPO_2_ values between the reused and nonreused plasters.

### Statistical Analysis

Data were collected using REDCap (Vanderbilt University, Nashville, TN, USA). Descriptive statistics are presented as median with interquartile range (IQR; 25th and 75th percentile) according to the data distribution. MitoPO_2_, signal quality, TcPO_2_, and ABI, are displayed as medians and IQRs at the fixed time points and locations, and trends in differences between time points and measurement locations are assessed in numbers and graphs. In addition, differences in pre- and postoperative values of mitoPO_2_ and TcPO_2_ were tested using the paired-sample *t*-test. p Values ≤0.05 were considered statistically significant.

## Results

A total of (52) patients were screened, with 10 patients meeting the inclusion criteria and being included in this study. The majority of the excluded patients (57.7%) did not qualify due to their use of medications known to induce photosensitivity, including antihypertensives and antidiabetic agents. Additionally, around 18% of screened patients could not be admitted a day earlier for placement of 5-ALA and were subsequently excluded. Participant characteristics are shown in Table 1. Five of the included patients had Rutherford stage 4, and 5 patients had Rutherford stage 5. The median ABI for the included patients was 0.52 (0.40–0.63) preoperatively and 0.73 (0.69–0.84) postoperatively.

**Table 1.** Characteristics of Patients.

Age (y)	72 (65–77.5)
Sex, n (%)	
Male	7 (70)
Female	3 (30)
Body mass index (kg/m^2^)	28.7 (20.8–29.3)
Smoking status, n (%)	
Smoker/Ex-smoker	3 (30)
Nonsmoker	7 (70)
Dyslipidemia, n (%)	8 (80)
Diabetes, n (%)	2 (20)
Hypertension, n (%)	5 (50)
History of coronary artery disease, n (%)	4 (40)
History of cerebrovascular disease, n (%)	1 (10)
COPD, n (%)	1 (10)
Renal dysfunction (eGFR <60 mL/min/1.73 m^2^), n (%)	1 (10)

Values are presented as median and interquartile range or count with percentage.

Abbreviations: COPD, chronic obstructive pulmonary disease; eGFR, estimated glomerular filtration rate.

### Treatment and Clinical Outcomes

A total of 11 limbs from 10 patients were treated, targeting 28 lesions predominantly located in the femoral, popliteal, and crural arteries. Most of these lesions (60%) underwent stent placement. Details regarding the revascularization therapies, along with information on the treated limbs and lesions, are presented in Table 2.

**Table 2.** Characteristics of Revascularization Therapy and Treated Limbs and Lesions.

Subject	Treatment	Treated arterial segments	Target lesions	GLASS: FP grading	GLASS: IP grading	Overall GLASS stage
#1	PTA and atherectomy	Popliteal and crural	Popliteal artery, ATA, peroneal artery	2	0	Stage II
#2	PTA and stent	Femoral, popliteal, and crural	SFA, popliteal artery, ATA, peroneal artery	4	4	Stage III
#3	PTA and stent	Femoral, popliteal, and crural	SFA, popliteal artery, ATA, posterior tibial artery	4	4	Stage III
#4	PTA with DEB and stent	Femoral	SFA	2	0	Stage II
#5	PTA and stent	Femoral, popliteal, and crural	SFA, popliteal artery, posterior tibial artery	2	3	Stage II
#6	PTA	Crural	Peroneal artery, ATA	0	4	Stage III
#7	PTA and stent	Iliac	CIA, EIA	4	4	Stage III
#8	PTA with DEB, atherectomy, and stent	Femoral, popliteal, and crural	SFA, popliteal artery, peroneal artery, ATA	4	0	Stage III
#9	PTA with DEB	Femoral-crural bypass	Femoral-ATA bypass	4	3	Stage III
#10	PTA with DEB and atherectomy	Femoral, popliteal, and crural	SFA, Popliteal artery, peroneal artery, ATA	4	4	Stage III

Abbreviations: ATA, anterior tibial artery; CIA, common iliac artery; DEB, drug-eluting balloon; EIA, external iliac artery; GLASS, Global Limb Anatomic Staging System; PTA, percutaneous transluminal angioplasty; SFA, superficial femoral artery.

Technical success of treatment was achieved in 100% of target lesion revascularization. Clinical improvement was assessed at the first follow-up visit at the outpatient clinic post-EVT, with a median time of 6.4 (5.4–7.1) weeks between the date of the procedure and the follow-up appointment. Clinical improvement was present in 7 patients (70%). Four patients with Rutherford class 4 (80%) improved clinically. Three patients with Rutherford class 5 (60%) demonstrated clinical improvement or reduced wound size.

### Tolerability of 5-ALA

No adverse events related to 5-ALA occurred in this study. Weak itching was reported by 1 patient during the 48 hours after plaster removal. However, the itch was tolerable and short-lived and did not necessitate intervention.

### Feasibility of mitoPO_2_ Measurements

All included patients could successfully undergo measurements using the COMET system at both the anterior tibialis and the lateral lower lateral leg locations. Measurements at the dorsum of the foot were not feasible. Due to the anatomy of the foot and the rigidity of the skin sensor, it was not possible to place the sensor there without introducing ambient light and applying external pressure. The signal quality of the obtained mitoPO_2_ measurements was sufficient. At the anterior tibialis, the median was 42 mmHg (35–50 mmHg) and 32 mmHg (30–41 mmHg) for the pre- and post-EVT measurements, respectively. At the lower lateral leg, the signal quality median was 44 mmHg (34–51 mmHg) and 39 mmHg (30–42 mmHg) for the pre- and post-EVT measurements, respectively. The application time of 12 hours was found to be sufficient. No problems occurred with the TcPO_2_ measurements at any of the 2 locations.

### Perfusion Measurements

#### Quantitative Pre- and Post-EVT Measurements

**Figure 4.**
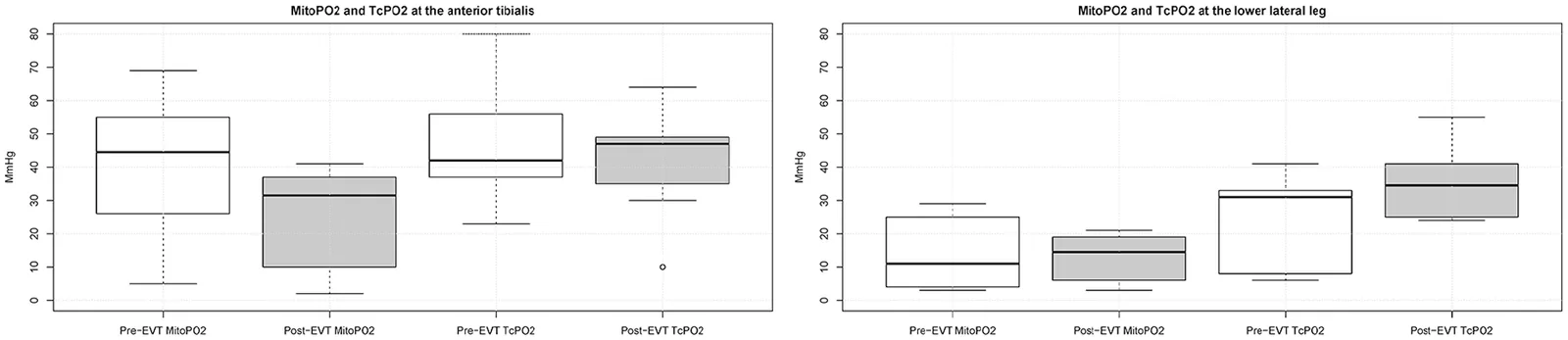
Box plots of mitoPO_2_ and TcPO_2_ at the anterior tibialis and lower lateral leg with changes pre- and post-EVT. EVT, endovascular therapy; mitoPO_2_, mitochondrial oxygen tension; TcPO_2_, transcutaneous oxygen pressure.

The individual values for mitoPO_2_ and TcPO_2_ along with clinical outcomes at the follow-up visit are presented in Table 3. Figure 4 displays the comparison in the median mitoPO_2_ and TcPO_2_ between pre- and post-EVT at the 2 measurement locations.

**Table 3.** MitoPO_2_ and TcPO_2_.

Subject	MitoPO_2_: Anterior tibialis			TcPO_2_: Anterior tibialis			Clinical improvement at follow-up
	Pre-EVT	Post-EVT	Change (%)	Pre-EVT	Post-EVT	Change (%)	
#1	43	37	−14	80	49	−39	Yes
#2^a^	65	26	−60	23	10	−57	Yes
#3	46	41	−11	42	55	31	Yes
#4	49	40	−18	60	48	−20	No
#5	55	37	−33	42	46	10	Yes
#6	16	2	−88	38	30	−21	Yes
#7	41	4	−90	47	64	36	Yes
#8	5	10	100	29	46	59	No
#9	69	37	−46	56	49	−13	No
#10^a^	26	10	−62	37	35	−5	Yes
Subject	MitoPO_2_: Lower lateral leg			TcPO_2_: Lower lateral leg			Clinical improvement at follow-up
	Pre-EVT	Post-EVT	Change (%)	Pre-EVT	Post-EVT	Change (%)	
#5	25	21	−16	33	32	−3	Yes
#6	8	10	25	30	37	23	Yes
#7	4	6	50	41	55	34	No
#8	3	3	0	6	24	300	No
#9	29	19	−35	32	25	−22	Yes
#10^a^	14	19	36	8	41	413	Yes

Abbreviations: EVT, endovascular therapy; mitoPO_2_, mitochondrial oxygen tension; TcPO_2_, transcutaneous oxygen pressure.

a Patients with diabetes.

The baseline median mitoPO_2_ was higher at the anterior tibialis [45 mmHg (30–54 mmHg)] than at the lower lateral leg [11 mmHg (5–22 mmHg)]. Post-EVT, median mitoPO_2_ [32 mmHg (10–37 mmHg)] significantly decreased at the anterior tibialis with a median change of [−40% (−62%−(−15%))]. At the lower lateral leg, no significant differences were detected pre- and post-EVT. Moreover, there was no significant difference in the trends between patients with and without clinical improvement.

Similar to mitoPO_2_, baseline TcPO_2_ was also higher at the anterior tibialis [42 mmHg (37–54 mmHg)] than at the lower lateral leg [31 mmHg (14–33 mmHg)]. No significant differences were detected post-EVT at either location. Furthermore, no correlation was found between TcPO_2_ and mitoPO_2_, pre- and post-EVT.

#### Plaster Reusability and mitoPO_2_ Measurements

MitoPO_2_ and signal quality in the post-EVT measurements were compared between the reused and nonreused plasters. Both at the anterior tibialis (n=9) and the lower lateral leg (n=6), median mitoPO_2_ and signal quality did not differ significantly, but on the re-used location the mitoPO_2_ value was slightly lower. Postoperative mitoPO_2_ at the tibia could not be obtained for 1 patient due to misplacement of the reused plaster.

## Discussion

This pilot study introduces a novel technique for measuring mitoPO_2_ as a measure of tissue perfusion in patients with PAD. Application of 5-ALA was found to be safe and tolerable in PAD patients. No systemic effects of 5-ALA were detected. MitoPO_2_ measurements were feasible at the tibia and at the lower lateral leg, but were not feasible at the dorsum of the foot. No adverse reactions or major discomfort was reported during the mitoPO_2_ measurements.

The COMET system has not been used in patients with PAD before. Our study cohort included patients with chronic limb-threatening ischemia, a condition marked by poor peripheral circulation, impaired wound healing, and a high risk of nonhealing ulcers. To ensure the safety of this frail group, we employed strict inclusion and exclusion criteria. Most exclusions were due to the use of medications that may induce photosensitivity, such as certain antihypertensives and antidiabetic agents. Although not formally contraindicated, they could increase the risk of phototoxic reactions in conventional photodynamic therapy (PDT).(Fukuda, Casas and Batlle, 2005). However, the light dose applied for mitoPO_2_ measurements (~1 J/cm²) is significantly lower than that used in PDT (~37 J/cm²), suggesting a substantially lower phototoxic risk. (Fukuda, Casas and Batlle, 2005)(Mik, Johannes and Zuurbier, 2008) Our findings confirmed the method’s safety and tolerability in this cohort, supporting the inclusion of patients on these medications in future trials, particularly considering that PAD patients often present with comorbidities such as diabetes and hypertension.

To ensure reliable signal quality, a 12-hour application time for the Alacare patch was chosen. This decision was based on the possibility that impaired microcirculation in CLTI may reduce the conversion of 5-ALA to PpIX, which is essential for generating a detectable signal. While the 12-hour duration was effective in this study, it introduced logistical barriers due to the need for hospital admission the day before the procedure. Notably, other studies measuring mitoPO_2_ were able to measure successfully after only 5 hours of application.^33^ Therefore, to improve clinical applicability future studies should determine whether shorter application times are also suitable for patients with PAD. Re-use of the plaster to compare postoperative values with preoperative values within an 8-hour timespan was feasible, but no definitive conclusions could be made due to the limited sample size. Another study investigating exercise-induced changes in mitoPO_2_ conducted multiple measurements for each patient and reapplied the plaster between the measurements with no problems.^27^ Although no conclusive evidence has been reported on the effect of temperature on mitoPO_2_ values,^33^ in our study, all measurements were taken at room temperature and skin temperature was checked using the COMET skin sensor and an infrared camera.

In this study, TcPO_2_ and MitoPO_2_ were measured on the same day as the EVT and showed a significant drop of perfusion postoperatively at the tibia. This is in accordance with an earlier study by Ma et al^34^ who investigated changes in tissue perfusion using hyperspectral and thermal imaging. This study showed that perfusion markers increased not earlier than 7 days post-EVT. Wagner et al^35^ and Gunnarsson et al^36^ also reported significant increase in TcPO_2_ perfusion at 6 and 10 weeks after EVT. Despite the limited number of studies investigating changes in tissue perfusion post-EVT, and the wide range of biomarkers investigated, results indicate that chronic ischemia and microvascular damage can impair actual tissue perfusion in the immediate period after EVT, and a new equilibrium in tissue perfusion may be achieved after 1 week.^37^ Therefore, it seems useful to perform post-EVT perfusion measurements at 1 to 2 weeks.

On the other hand, it is still unknown how trends in mitoPO_2_ are correlated with oxygen supply and tissue regeneration, and how they will change over time in these patients. Physiologically, mitoPO_2_ reflects the intracellular balance between oxygen supply and consumption.^38^ In healthy tissue, increased metabolic demand leads to a rapid breakdown of adenosine triphosphate and the subsequent release of vasodilators, which lower mitoPO_2_ while enhancing blood flow.^39,40^ The postoperative decrease in mitoPO_2_ that was measured in this study could be the result of increased cellular metabolism and might therefore indicate healing of affected tissue.^33^ MitoPO_2_ and TcPO_2_ values were lower at the lower lateral leg compared to the anterior tibialis muscle, which indicates that lower parts of the leg are generally less perfused in PAD patients. Considering the different values for the measurement locations, and the high variation between patients, mitochondrial oxygenation should always be compared at the same location and the patient should serve as their own reference.

MitoPO_2_’s sensitivity to changes in physiologic decompensation makes the COMET device a potentially valuable instrument to use in clinical care of PAD patients. However, a significant limitation is the COMET skin sensor’s design. A flat surface area is required that can accommodate the skin sensor without introducing ambient light or pressure. This prevented us from obtaining measurements at the dorsum of the foot. We also considered using the plantar side of the foot, but decided against it due to its thick keratinized epidermis which could affect PpIX accumulation and fluorescence signal detection. In addition, applying pressure on the sensor should be avoided unless it is intentional to induce obstruction of flow, which is challenging to achieve on weight-bearing surfaces like the plantar foot. The inability to measure distally is significant for patients with advanced distal disease.

Current methods for measuring tissue oxygenation focus mainly on systemic hemodynamic parameters, but these methods are not always indicative of early tissue hypoxia.^41,42^ Monitoring mitoPO_2_ can provide insights into tissue oxygenation and potential hypoxia during surgical procedures,^33^ offering a more direct measure of cellular oxygenation than traditional systemic hemodynamic parameters. Post-EVT assessment of tissue perfusion is not conducted regularly.^34^ However, the Global Vascular Guidelines emphasize on the relevance of including tissue perfusion measurements in PAD to predict tissue regeneration and to determine the necessary next steps.^5,43–46^

This study has several limitations. The strict exclusion criteria and complex in this tolerability study posed constraints on the recruitment process, resulting in a small sample size which made it difficult to generalize conclusions. Additionally, the study could not confirm whether the observed changes in mitoPO_2_ could predict clinical improvement due to the absence of long-term clinical follow-up. Finally, due to the learning curve and sensitivity of the device, we were not able to consistently measure mitoVO_2_ and mitoDO_2_ in this cohort. Future studies should aim to establish mitoPO_2_’s clinical relevance, define physiological thresholds, and correlate its values with patient outcomes. In addition, future protocols should investigate the feasibility of employing less strict criteria, and shorter Alacare application time. Future improvements in sensor design and measurement protocols could ultimately expand its utility in advanced PAD management.

## Conclusion

This pilot study demonstrates that patients with PAD can safely undergo a test with 5-ALA and that mitoPO_2_ measurements are feasible at the tibia and lower lateral leg. A significant decrease in mitoPO_2_ was detected at the tibia of patients undergoing EVT. Further research is required with less stringent exclusion criteria to determine optimal application times. Furthermore, future studies should include more patients with longer follow-up to investigate the relationship between mitoPO_2_, oxygen supply and tissue regeneration.
